# *Ixodes scapularis* Tick Cells Control *Anaplasma phagocytophilum* Infection by Increasing the Synthesis of Phosphoenolpyruvate from Tyrosine

**DOI:** 10.3389/fcimb.2017.00375

**Published:** 2017-08-17

**Authors:** Alejandro Cabezas-Cruz, Pedro J. Espinosa, Dasiel A. Obregón, Pilar Alberdi, José de la Fuente

**Affiliations:** ^1^Biologie Moléculaire et Immunologie Parasitaires (BIPAR), Unité Mixte de Recherche (UMR), Institut National Recherche Agronomique, Agence Nationale Sécurité Sanitaire Alimentaire Nationale (ANSES), Ecole Nationale Vétérinaire d'Alfort, Université Paris-Est Maisons-Alfort, France; ^2^Department of Parasitology, Faculty of Science, University of South Bohemia České Budějovice, Czechia; ^3^Institute of Parasitology, Biology Center, Czech Academy of Sciences České Budějovice, Czechia; ^4^SaBio, Instituto de Investigación en Recursos Cinegéticos IREC (CSIC-UCLM-JCCM) Ciudad Real, Spain; ^5^Cell and Molecular Biology Laboratory, University of Sao Paulo Sao Paulo, Brazil; ^6^Department of Veterinary Pathobiology, Center for Veterinary Health Sciences, Oklahoma State University Stillwater, OK, United States

**Keywords:** proteomics, transcriptomics, phosphoenolpyruvate, glycerol- 3-phosphate, *Ixodes scapularis*, *Anaplasma phagocytophilum*

## Abstract

The obligate intracellular pathogen, *Anaplasma phagocytophilum*, is the causative agent of life-threatening diseases in humans and animals. *A. phagocytophilum* is an emerging tick-borne pathogen in the United States, Europe, Africa and Asia, with increasing numbers of infected people and animals every year. It is increasingly recognized that intracellular pathogens modify host cell metabolic pathways to increase infection and transmission in both vertebrate and invertebrate hosts. Recent reports have shown that amino acids are central to the host–pathogen metabolic interaction. In this study, a genome-wide search for components of amino acid metabolic pathways was performed in *Ixodes scapularis*, the main tick vector of *A. phagocytophilum* in the United States, for which the genome was recently published. The enzymes involved in the synthesis and degradation pathways of the twenty amino acids were identified. Then, the available transcriptomics and proteomics data was used to characterize the mRNA and protein levels of *I. scapularis* amino acid metabolic pathway components in response to *A. phagocytophilum* infection of tick tissues and ISE6 tick cells. Our analysis was focused on the interplay between carbohydrate and amino acid metabolism during *A. phagocytophilum* infection in ISE6 cells. The results showed that tick cells increase the synthesis of phosphoenolpyruvate (PEP) from tyrosine to control *A. phagocytophilum* infection. Metabolic pathway analysis suggested that this is achieved by (i) increasing the transcript and protein levels of mitochondrial phosphoenolpyruvate carboxykinase (PEPCK-M), (ii) shunting tyrosine into the tricarboxylic acid (TCA) cycle to increase fumarate and oxaloacetate which will be converted into PEP by PEPCK-M, and (iii) blocking all the pathways that use PEP downstream gluconeogenesis (i.e., *de novo* serine synthesis pathway (SSP), glyceroneogenesis and gluconeogenesis). While sequestering host PEP may be critical for this bacterium because it cannot actively carry out glycolysis to produce PEP, excess of this metabolite may be toxic for *A. phagocytophilum*. The present work provides a more comprehensive view of the major amino acid metabolic pathways involved in the response to pathogen infection in ticks, and provides the basis for further studies to develop novel strategies for the control of granulocytic anaplasmosis.

## Introduction

*Anaplasma phagocytophilum* (Rickettsiales: Anaplasmataceae) is an obligate intracellular bacterium that produces life-threatening disease in humans and animals (Kocan et al., 2015). This pathogen is mainly transmitted by *Ixodes* spp. ticks in the United States, Europe, Africa and Asia (de la Fuente et al., 2008; Kocan et al., 2015). *A. phagocytophilum* infects vertebrate host granulocytes, and tick midgut, hemocytes and salivary glands (de la Fuente et al., 2008; Stuen et al., 2013; Kocan et al., 2015). The life cycle of *A. phagocytophilum* includes two morphological forms, the dense and reticulated cells, which are the infective and replicative stages of this bacterium, respectively (Stuen et al., 2013; Kocan et al., 2015). *A. phagocytophilum* has a very small genome (approximately 1.47 Mb) with a reduced number of effector proteins (Dunning et al., 2006; Sinclair et al., 2014, 2015). Therefore, as an evolutionary adaptation to its multi-host life style, this pathogen uses similar strategies to manipulate host cells and facilitate infection in vertebrates and ticks (Stuen et al., 2013; de la Fuente et al., 2016a). These mechanisms include but are not limited to remodeling of the cytoskeleton, inhibition of cell apoptosis, manipulation of the immune response, and modification of cell epigenetics and metabolism (Cabezas-Cruz et al., 2016, 2017a,b; de la Fuente et al., 2016a).

Host metabolism manipulation by bacteria has deep evolutionary roots. Not only pathogens have been shown to manipulate host metabolism, but commensal bacteria can also induce dramatic changes in host physiology (and even behavior) by affecting host metabolism (Olive and Sassetti, 2016; Leitão-Gonçalves et al., 2017). Thus, host-bacteria associations might be based on both transient and lasting metabolic cooperation and competition (Husnik et al., 2013; Zhang and Rubin, 2013). By transiently exploiting host metabolism, bacterial pathogens often fall within the first category (Zhang and Rubin, 2013; Olive and Sassetti, 2016). There is evidence that bacterial pathogens rely on host amino acid metabolism and in response the host “starves” the pathogen by “denying” the required amino acids (Zhang and Rubin, 2013). For example, it has been shown that *Mycobacterium tuberculosis* as well as *Salmonella* amino acid auxotroph strains are attenuated *in vivo* (O'Callaghan et al., 1988; Hondalus et al., 2000). Pathogens respond to this host-mediated amino acid starvation in different ways: (i) by differentiating to a viable but non-replicating form (e.g., *Chlamydia trachomatis*), (ii) by constitutively synthesizing their own amino acids (e.g., *M. tuberculosis*) and (iii) by manipulating host cell machinery to make amino acids available to the bacteria (e.g., *Legionella pneumophila*) (Zhang and Rubin, 2013). In the case of vector-borne bacteria, such as *A. phagocytophilum*, a solely-competition-based mechanism does not explain how this vector-pathogen ensemble is kept during evolution. Therefore, alternative models of vector-pathogen interaction, where both partners benefit from the association, should be explored (de la Fuente et al., 2016b; Cabezas-Cruz et al., 2017c).

Out of twenty amino acids, the *A. phagocytophilum* genome encodes only for the enzymes responsible of proline, glutamine, glycine and aspartate biosynthesis (Dunning et al., 2006). Like other *Rickettsia* spp., this intracellular pathogen cannot actively carry out glycolysis (Dunning et al., 2006). The glycolysis enzymes present are reduced to those that produce glyceraldehyde-3-phosphate and dihydroxyacetone phosphate (DHAP) from phosphoenolpyruvate (PEP). This fact suggests that *A. phagocytophilum* may hijack some glycolytic intermediates produced by the host to complement its limited metabolic capacity. Recently, transcriptomics, proteomics and metabolomics analyses of infected *I. scapularis* ISE6 cells showed that *A. phagocytophilum* infection affects amino acid and carbohydrate metabolic pathways (Villar et al., 2015; Cabezas-Cruz et al., 2017a). These results suggested that *A. phagocytophilum* subverts amino acid and carbohydrate metabolism to facilitate infection and multiplication in tick cells. This evidence led us to the hypothesis that *A. phagocytophilum* infection subverts amino acid and carbohydrate metabolism simultaneously to increase the levels of and hijack PEP, which is the glycolytic intermediate with the highest-energy phosphate bond found in living organisms (Berg et al., 2002).

To test this hypothesis, the metabolism of the 20 amino acids was characterized in the tick vector *I. scapularis* in response to *A. phagocytophilum* infection. Firstly, the composition of the 20 amino acid metabolic pathways was annotated using the recently published genome of *I. scapularis* (de la Fuente et al., 2016c; Gulia-Nuss et al., 2016). Then, previously published transcriptomics and proteomics data (Ayllón et al., 2015; Villar et al., 2015) was used to characterize the mRNA and protein levels of amino acid metabolic pathway components in response to *A. phagocytophilum* infection of *I. scapularis* nymphs, female midguts and salivary glands, and ISE6 cells. Metabolic pathways analysis combined with quantitative metabolomics suggested a mechanism by which *A. phagocytophilum* infection increases the intracellular concentration of PEP which in turn control bacterial burden. The results also showed that the increase in PEP levels is achieved by using tyrosine as a carbon source via tricarboxylic acid (TCA) cycle. These results expanded our knowledge of the different pathways affected by *A. phagocytophilum* infection in ticks, and provided new potential targets for the development of therapeutic and prevention strategies for the control of human granulocytic anaplasmosis and other diseases caused by *Rickettsia* spp. that may use similar mechanisms for infection of the tick vector.

## Materials and methods

### Annotation of the amino acid metabolic pathway components in the *I. scapularis* genome

The *I. scapularis* genome (Gulia-Nuss et al., 2016) was searched with the specific names of genes encoding for enzymes involved in the 20 amino acid metabolic pathways. When records were not obtained using specific enzyme names, then the *I. scapularis* genome was searched with the Blastp tool from the Basic Local Alignment Search Tool (BLAST) using the human ortholog as “query” (Altschul et al., 1990; Madden et al., 1996). The sequences with the lowest *E*-value were selected. The conserved domains of identified protein sequences were classified using the protein families database Pfam (Finn et al., 2014). The *I. scapularis* orthologs found in the genome were double-checked by searching the National Center for Biotechnology Information (NCBI) databases using as queries the tick homologs identified in the previous step and excluding “*I. scapularis*” genome database from the search.

### Characterization of the *I. scapularis* mRNA and protein levels in response to *A. phagocytophilum* infection

The quantitative transcriptomics and proteomics data for uninfected and *A. phagocytophilum*-infected *I. scapularis* nymphs, female midguts and salivary glands, and ISE6 cultured cells were obtained from previously published results (Ayllón et al., 2015; Villar et al., 2015) and deposited at the Dryad repository database, NCBI's Gene Expression Omnibus database and ProteomeXchange Consortium via the PRIDE partner repository with the dataset identifier PXD002181 and doi: 10.6019/PXD002181. The identified genes in the amino acid metabolic pathways were searched against the transcriptomics and proteomics data to characterize their mRNA and protein levels in response to *A. phagocytophilum* infection.

### *Ixodes scapularis* ISE6 cells

The *I. scapularis* embryo-derived tick cell line ISE6, provided by Ulrike Munderloh, University of Minnesota, USA, was cultured in L-15B300 medium as described previously (Munderloh et al., 1994), except that the osmotic pressure was lowered by the addition of one-fourth sterile water by volume. The ISE6 cells were first inoculated with *A. phagocytophilum* (human NY18 isolate)-infected HL-60 cells (de la Fuente et al., 2005) and maintained according to Munderloh et al. (1999). Pathogen manipulation and disposal of residuals were performed following biosafety level-2 (BSL2) laboratory procedures.

### Determination of glycerol-3-phosphate and phosphoenolpyruvate levels

ISE6 tick cells (approximately 5 × 10^5^ cells/well) were inoculated with *A. phagocytophilum* NY18 then sampled at 7 days post-infection (dpi, % infected cells > 70%). Uninfected cells were included as controls. Harvested cells were used to determine the concentration of glycerol 3-phosphate (G-3P) and PEP using the glycerol 3-phosphate colorimetric assay Kit (Sigma Cat. No. MAK207) or the PEP colorimetric/fluorometric assay kit (Sigma Cat. No. MAK102) respectively, following manufacturer's protocols. G-3P and PEP levels (ng/μl) were compared between untreated and treated cells by Student's *t*-test with unequal variance (*P* < 0.05; *N* = 4 biological replicates).

### Pharmacological studies in cultured tick cells

Uninfected ISE6 tick cells were treated for 6 h with 1 μM Nitisinone (Sigma Cat. No. SML0269) to inhibit the activity of hydroxyphenylpyruvate dioxygenase (HPPD). Then, they were infected with *A. phagocytophilum* NY18. Cells were harvested at 24 and 72 h and used for Annexin V-FITC staining to detect cell apoptosis (see below), for DNA extraction to quantify the levels of with *A. phagocytophilum* (see below) and to determine the levels of PEP as described above. All treatments were done in quadruplicate. Uninfected and infected untreated cells were used as controls.

### Determination of *A. phagocytophilum* burden by real-time PCR

*Anaplasma phagocytophilum* NY18 DNA levels were characterized by *msp4* real-time PCR normalizing against tick ribosomal protein S4 (*rps4*) as described previously (Alberdi et al., 2015). Normalized Ct values were compared between untreated and treated cells by Student's *t*-test with unequal variance (*P* < 0.05; *N* = 4).

### Annexin V-FITC staining to detect cell apoptosis after experimental infection with *A. phagocytophilum*

Approximately 5 × 10^5^–1 × 10^6^ uninfected and *A. phagocytophilum*-infected ISE6 tick cells were collected after different treatments. Apoptosis was measured by flow cytometry using the Annexin V-fluorescein isothiocyanate (FITC) apoptosis detection kit (Immunostep, Salamanca, Spain) following the manufacturers protocols. The technique detects changes in phospholipid symmetry analyzed by measuring Annexin V (labeled with FITC) binding to phosphatidylserine, which is exposed in the external surface of the cell membrane in apoptotic cells. Cells were stained simultaneously with the non-vital dye propidium iodide (PI) allowing the discrimination of intact cells (Annexin V-FITC negative, PI negative) and early apoptotic cells (Annexin V-FITC positive, PI negative). All samples were analyzed on a FAC-Scalibur flow cytometer equipped with CellQuest Pro software (BD Bio-Sciences, Madrid, Spain). The viable cell population was gated according to forward-scatter and side-scatter parameters. The percentage of apoptotic cells was determined by flow cytometry after Annexin V-FITC and PI labeling and compared between treated and untreated uninfected cells by Student's *t*-test with unequal variance (*P* < 0.05; *N* = 4).

## Results

### Major amino acid metabolic pathways described in model organisms are present in *I. scapularis* and are affected by *A. phagocytophilum* infection

**Figure 1:**
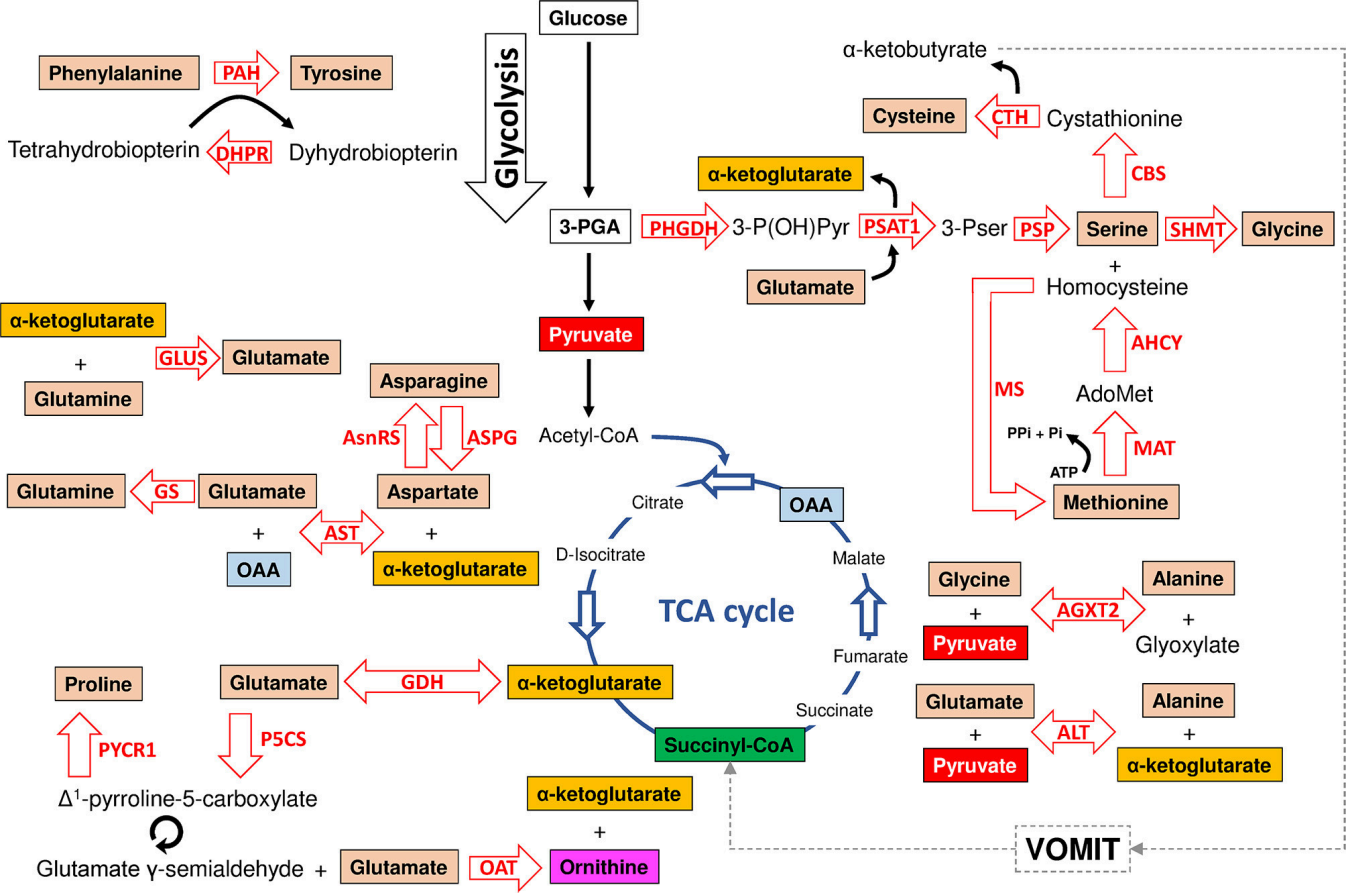
Model of amino acid synthesis in *I. scapularis*. The main enzymes involved in tyrosine, cysteine, serine, glycine, methionine, alanine, glycine, glutamate, glutamine, asparagine, aspartate and proline synthesis that were identified in the genome of *I. scapularis* (Table 1) are shown. The interplay between amino acid and glucose metabolism (i.e., glycolysis and TCA cycle) intermediates is also shown. The names of the enzymes were abbreviated as follow: phenylalanine hydroxylase (PAH), dihydropteridine reductase (DHPR), glutamate synthase (GLUS), asparagine synthetase (AsnRS), asparaginase (ASPG), glutamine synthetase (GS), aspartate aminotransferase (AST), glutamate dehydrogenase (GDH), pyrroline-5-carboxylate reductase (PYCR1), delta-1-pyrroline-5-carboxylate synthetase (P5CS), ornithine aminotransferase (OAT), alanine transaminase (ALT), alanine-glyoxylate aminotransferase 2 (AGXT2), methionine adenosyltransferase (MAT), methionine synthase (MS), Adenosylhomocysteinase (AHCY), phosphoserine phosphatase (PSP), serine hydroxymethyltransferase (SHMT), cystathionine beta-synthase (CBS), cystathionase (Cystationine gamma lyase) (CTH), phosphoserine aminotransferase (PSAT1) and 3-phosphoglycerate dehydrogenase (PHGDH). The metabolic intermediates were abbreviated as follow: oxaloacetate (OAA), 3-phosphoglycerate (3-PGA), 3-phosphohydroxypyruvate (3-P(OH)Pyr), 3-phosphoserine (3-Pser), inorganic phosphate (Pi), adenosine triphosphate (ATP) and S-adenosylmethionine (AdoMet). The dashed line represents the VOMIT pathway (where V stands for valine, O for odd-chain fatty acids, M for methionine, I for isoleucine, and T for threonine) that transforms α-ketobutyrate in the TCA cycle intermediate succinyl-CoA. The VOMIT pathway involves three enzymes (propionyl-CoA carboxylase beta chain, methylmalonyl-CoA epimerase and methylmalonyl-CoA mutase) no displayed in the figure (Table 1). Glutamate γ-semialdehyde is the open-chain tautomer of Δ^1^-pyrroline-5-carboxylate. The circular arrow symbol represents the nonenzymatic and reversible interconversion of Δ^1^-pyrroline-5-carboxylate to glutamate γ-semialdehyde.

**Figure 2:**
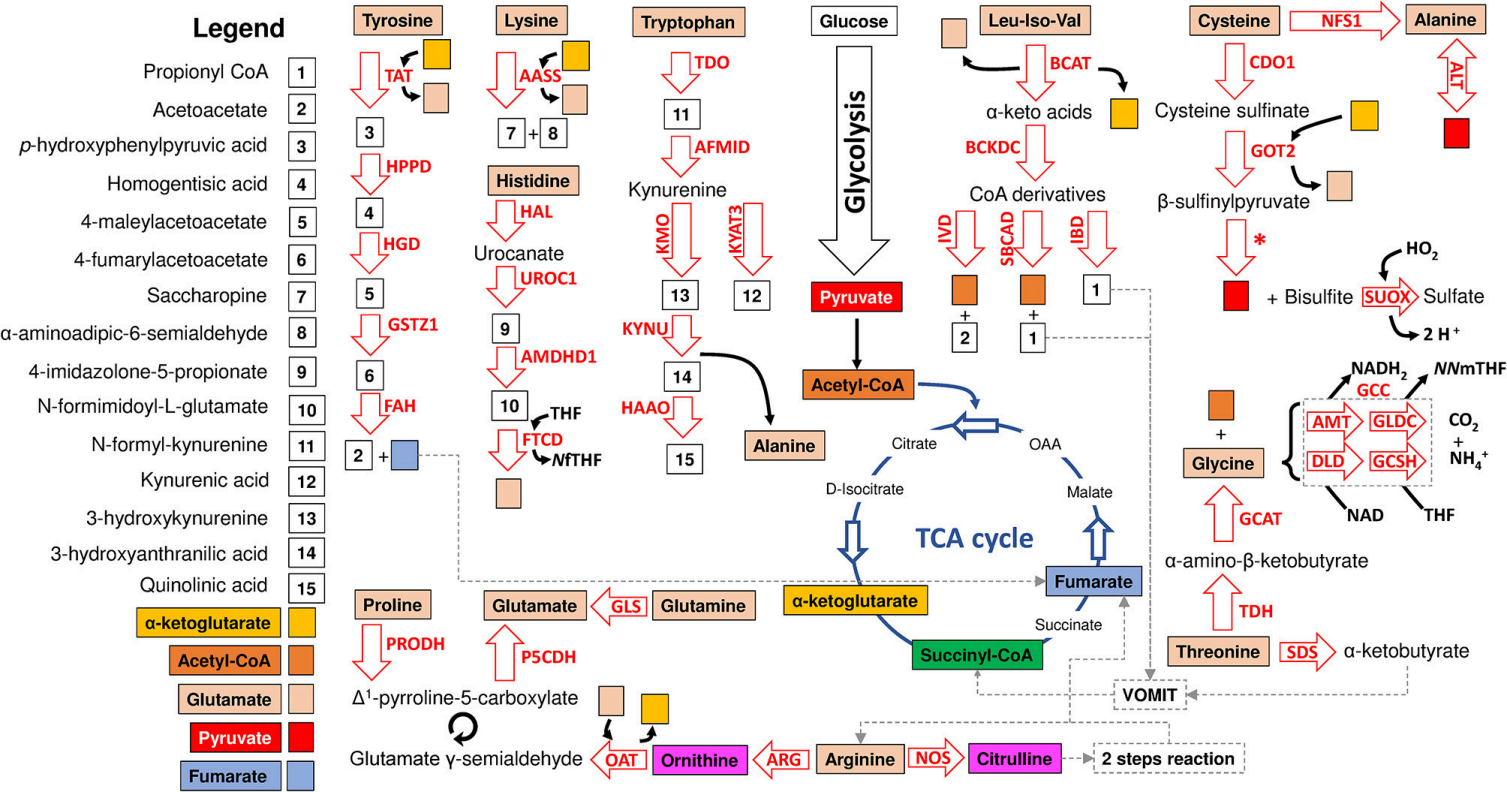
Model of amino acid degradation in *I. scapularis*. The main enzymes involved in tyrosine, cysteine, glycine, alanine, glycine, glutamate, glutamine, arginine, lysine, tryptophan, histidine, threonine, proline, leucine (Leu), isoleucine (Iso) and valine (Val) degradation and identified in the genome of *I. scapularis* (Table 1) are shown. The interplay between amino acid and glucose metabolism (i.e., glycolysis and TCA cycle) intermediates is also shown. The names of the enzymes were abbreviated as follow: tyrosine aminotransferase (TAT), hydroxyphenylpyruvate dioxygenase (HPPD), homogentisate 1,2-dioxygenase (HGD), maleylacetoacetate isomerase (GSTZ1), fumarylacetoacetate hydrolase (FAH), α-aminoadipic semialdehyde synthase (AASS), histidine ammonia-lyase (HAL), urocanate hydratase 1 (UROC1), imidazolone propionase (AMDHD1), formiminotransferase cyclodeaminase (FTCD), tryptophan 2,3-dioxygenase (TDO), kynurenine formamidase (AFMID), kynurenine 3-monooxygenase (KMO), kynurenine-oxoglutarate transaminase 3 (KYAT3), kynureninase (KYNU), 3-hydroxyanthranilate 3,4-dioxygenase (HAAO), branched-chain amino acid aminotransferase (BCAT), isovaleryl-CoA dehydrogenase (IVD), short/branched-chain acyl-CoA dehydrogenase (SBCAD), isobutyryl-CoA dehydrogenase (IBD), cysteine dioxygenase 1 (CDO1), GOT2 aspartate aminotransferase (GOT2), cysteine desulfurase (NFS1), alanine transaminase (ALT), sulfite oxidase (SUOX), glycine cleavage complex (GCC) which includes four enzymes: aminomethyltransferase (AMT), glycine dehydrogenase (GLDC), dihydrolipoamide dehydrogenase (DLD) and glycine cleavage system H protein (GCSH), 2-amino-3-ketobutyrate coenzyme A ligase (GCAT), threonine dehydrogenase (TDH), L-serine dehydratase/L-threonine deaminase (SDS), glutaminase (GLS), proline dehydrogenase (PRODH), delta-1-pyrroline-5-carboxylate dehydrogenase (P5CDH), ornithine aminotransferase (OAT), arginase (ARG), nitric oxide synthase (NOS) and branched-chain α-ketoacid dehydrogenase complex (BCKDC) which includes three enzymes (branched-chain α-keto acid decarboxylase, lipoamide acyltransferase and dihydrolipoamide dehydrogenase) no displayed in the figure (Table 1). Citrulline is transformed in arginine by a “two-step reaction” including argininosuccinate synthetase (ASS) and argininosuccinate lyase (ASL) which are not displayed in the figure but are available in Table 1. The metabolic intermediates were abbreviated as follow: oxaloacetate (OAA), N^5^-N^10^-methylene tetrahydrofolate (*NN*mTHF), 5-formiminotetrahydrofolate (*N*fTHF) and tetrahydrofolate (THF). The VOMIT pathway (where V stands for valine, O for odd-chain fatty acids, M for methionine, I for isoleucine, and T for threonine) enzymes transform α-ketobutyrate and propionyl CoA into the TCA cycle intermediate succinyl-CoA. The VOMIT pathway enzymes (propionyl-CoA carboxylase beta chain, methylmalonyl-CoA epimerase and methylmalonyl-CoA mutase) are not displayed in the figure but are available in Table 1. Glutamate γ-semialdehyde is the open-chain tautomer of Δ^1^-pyrroline-5-carboxylate. The circular arrow symbol represents the nonenzymatic and reversible interconversion of Δ^1^-pyrroline-5-carboxylate to glutamate γ-semialdehyde. The asterisk shows the position of a desulfurization reaction.

The major synthesis and degradation pathways of the 20 amino acids were selected for characterization. A total of 72 genes coding for the proteins involved in the metabolism of the major 20 amino acids were identified in the *I. scapularis* genome (Table 1). Based on these results, models for amino acid synthesis of nonessential amino acids and degradation in ticks were proposed (Figures 1, 2). Despite being hematophagous ectoparasites that ingest large amounts of a protein-rich diet, the tick genome contains all genes coding for enzymes responsible for major amino acid metabolic pathways found in animal models (Berg et al., 2002). An exception was the enzyme cysteine sulfinic acid decarboxylase (CSAD), involved in cysteine metabolism, and for which no orthologue was found in the *I. scapularis* genome. At least in humans, cysteine sulfinate can be transformed to hypotaurine by CSAD. Hypotaurine is oxidased nonenzymatically to taurine. Searches in sequence databases of other tick species did not produce a CSAD orthologue.

**Table 1:** Annotation of amino acid metabolism enzymes identified in the genome of *Ixodes scapularis*.

**Amino acid/Name**	**Abbreviation**	**Genome accession**	**Uniprot**
**ALANINE**			
Alanine transaminase 1	ALT1	ISCW013629	B7QLI8
Alanine transaminase 2	ALT2	ISCW019179	B7PLP0
**CYSTEINE**			
Cystathionine beta-synthase	CBS	ISCW003755	B7PF92
Cysteine dioxygenase 1	CDO1	ISCW008991	B7PY67
Cysteine desulfurase	NFS1	ISCW004705	B7PGB2
Cystathionase (Cystationine gamma Lyase)	CTH	ISCW007691	B7PU74
Methionine adenosyltransferase	MAT	ISCW004274	B7PJM6
Adenosylhomocysteinase 1	AHCY1	ISCW015944	B7P4Y1
Adenosylhomocysteinase 2	AHCY2	ISCW016595	B7PCR2
Sulfite oxidase	SUOX	ISCW004861	B7PGM8
**ASPARTIC ACID (ASPARTATE)**			
Aspartate aminotransferase	AST	ISCW014718	B7QLB0
GOT2 aspartate aminotransferase (mitochondrial isoenzyme)	GOT2	ISCW020516	B7Q1F6
Asparaginase	ASPG	ISCW022371	B7QGF3
**GLUTAMIC ACID (GLUTAMATE)**			
Glutamate synthase	GLUS	ISCW005873	B7PNR9
Glutamate dehydrogenase	GDH	ISCW000393	B7P4E1
**PHENYLALANINE**			
Phenylalanine hydroxylase	PAH	ISCW006510	B7PLQ7
**GLYCINE**			
Aminomethyltransferase	AMT [GCC, T-subunit]	ISCW001092	B7P6X5
Glycine dehydrogenase	GLDC [GCC, P-subunit]	ISCW006033	B7PR45
Dihydrolipoamide dehydrogenase	DLD [GCC, L-subunit]	ISCW001462	B7P465
Glycine cleavage system H protein	GCSH [GCC, H-subunit]	ISCW002099	B7P916
Glycine/Serine hydroxymethyltransferase	SHMT	ISCW004246	B7PGD5
Alanine-glyoxylate aminotransferase 2	AGXT2	ISCW020823	B7Q1E3
**HISTIDINE**			
Urocanate hydratase 1	UROC1	ISCW021677	B7Q456
Histidine ammonia-lyase	HAL	ISCW012913	B7QBL3
Imidazolone propionase	AMDHD1	ISCW012713	B7QD45
Formiminotransferase cyclodeaminase	FTCD	ISCW010907	B7Q6E5
**ISOLEUCINE, LEUCINE, VALINE**			
Branched-chain amino acid aminotransferase	BCAT	ISCW015784	B7P5K8
Branched-chain α-keto acid decarboxylase	(BCKDHA) [BCKDC, E1]	ISCW018615	B7PL04
Lipoamide acyltransferase	(DBT) [BCKDC, E2]	ISCW018104	B7PEH7
Dihydrolipoamide dehydrogenase	(DLD) [BCKDC, E3]	ISCW001462	B7P465
short/branched-chain acyl-CoA dehydrogenase	(SBCAD) [Only for Isoleucine]	ISCW002863	B7P7P2
Isovaleryl-CoA dehydrogenase	(IVD) [Only for Leucine]	ISCW000209	B7P6T5
Isobutyryl-CoA dehydrogenase	(IBD) [Only for Valine]	ISCW003911	B7PJR8
**LYSINE**			
α-aminoadipic semialdehyde synthase	AASS	ISCW008489	B7PT93
**METHIONINE**			
Propionyl-CoA carboxylase beta chain	PCCB [VOMIT]	ISCW010503	B7Q9C9
Methylmalonyl-CoA epimerase	MCEE [VOMIT]	ISCW003889	B7PF37
Methylmalonyl-CoA mutase	MUT [VOMIT]	ISCW020522	B7Q1G1
Methionine synthase	MS	ISCW020738	B7Q2S9
**ASPARAGINE**			
Asparagine synthetase	AsnRS	ISCW004505	B7PH83
**PROLINE**			
Delta-1-pyrroline-5-carboxylate synthetase	P5CS	ISCW005278	B7PPZ5
Proline dehydrogenase	PRODH	ISCW000787	B7P4N1
Ornithine aminotransferase 1	OAT1	ISCW024076	B7P713
Ornithine aminotransferase 2	OAT2	ISCW007128	B7PUI7
Ornithine aminotransferase 3	OAT3	ISCW023390	B7QHR2
Pyrroline-5-carboxylate reductase A	PYCR1A	ISCW021802	B7Q8R2
Pyrroline-5-carboxylate reductase B	PYCR1B	ISCW020302	B7Q351
**GLUTAMINE**			
Glutamine synthetase	GS	ISCW018771	B7PNU5
Glutaminase	GLS	ISCW020515	B7Q1F5
**ARGININE**			
Argininosuccinate synthase	ASS	ISCW017094	B7PA12
Delta-1-pyrroline-5-carboxylate dehydrogenase	P5CDH	ISCW015982	B7P0M7
Argininosuccinate lyase	ASL	ISCW009296	B7Q096
Arginase	ARG	ISCW014441	B7QIY4
Nitric oxide synthase	NOS	ISCW018074	B7PGS0
**SERINE**			
3-phosphoglycerate dehydrogenase	PHGDH	ISCW015967	B7P3M8
Phosphoserine aminotransferase	PSAT1	ISCW020198	B7Q0D6
Phosphoserine phosphatase	PSP	ISCW003612	B7PHY5
**THREONINE**			
Threonine dehydrogenase	TDH	ISCW020011	B7PZ94
L-serine dehydratase/L-threonine deaminase	SDS	ISCW005673	B7PN84
2-amino-3-ketobutyrate coenzyme A ligase	GCAT	ISCW015295	B7QMM1
**TRYPTOPHAN**			
Tryptophan 2,3-dioxygenase	TDO	ISCW024183	B7PBJ2
Kynurenine formamidase	AFMID	ISCW007881	B7PSM5
Kynurenine-oxoglutarate transaminase 3 A	KYAT3-A	ISCW012664	B7QG90
Kynurenine-oxoglutarate transaminase 3 B	KYAT3-B	ISCW012663	B7QG89
Kynurenine 3-monooxygenase	KMO	ISCW011595	B7Q540
Kynureninase A	KYNU-A	ISCW012597	B7QAC8
Kynureninase B	KYNU-B	ISCW005441	B7PL61
3-hydroxyanthranilate 3,4-dioxygenase	HAAO	ISCW007038	B7PU50
**TYROSINE**			
Dihydropteridine reductase	DHPR	ISCW022552	B7QAP3
Tyrosine aminotransferase	TAT	ISCW014463	B7QLV1
Hydroxyphenylpyruvate dioxygenase	HPPD	ISCW018238	B7PHP6
Homogentisate 1,2-dioxygenase	HGD	ISCW004334	B7PK05
Maleylacetoacetate isomerase	GSTZ1	ISCW023198	B7QMI2
Fumarylacetoacetate hydrolase	FAH	ISCW020196	B7Q0D4

There is a major crosstalk between carbohydrate and amino acid metabolism. For example, the α-ketoacids, α-ketoglutarate, oxaloacetate (OAA), and pyruvate, can be converted into amino acids in one step through the addition of an amino group (Berg et al., 2002). In this group of amino acids are alanine, aspartate and glutamate that can be synthesized from pyruvate, OAA and α-ketoglutarate, respectively. All enzymes required for these transformations were found in the *I. scapularis* genome (Figure 1). Aspartate can also be formed by deamination of asparagine catalyzed by asparaginase (ASPG). As displayed in Figure 1, glutamate and aspartate can be transformed into glutamine and asparagine in amidation reactions catalyzed by glutamine synthetase (GS) and asparagine synthetase (AsnRS), respectively. Apart from glutamine, glutamate is the precursor of two other nonessential amino acids: proline and arginine. The enzyme Δ-1-pyrroline-5-carboxylate synthetase (P5CS) converts glutamate to glutamate γ-semialdehyde, an intermediate in the biosynthesis of proline (Figure 1), ornithine and arginine (Figure 2). Although the metabolism of ornithine and citruline was not considered in this study, these amino acids were included whenever they form part of the metabolism of the 20 amino acids. Another major contributor to amino acid synthesis is the glycolytic intermediate 3-phosphoglycerate (3-PGA), which is the precursor of serine, cysteine, and glycine (Figure 1). Finally, tyrosine can be synthesized from the essential amino acid phenylalanine by action of the enzyme phenylalanine hydroxylase (PAH). The reaction catalyzed by PAH requires a cofactor, tetrahydrobiopterin, which is maintained in the reduced state by the NADH-dependent enzyme, dihydropteridine reductase (DHPR). Both enzymes were found in the *I. scapularis* genome.

In comparison to synthesis, amino acid degradation is carried out by far more complicated reactions requiring a larger number of steps (Figure 2). However, likewise synthesis, amino acid degradation is tightly connected to carbohydrate metabolism. Thus, depending on their metabolic fate, amino acids are classified in glucogenic (aspartate, asparagine, alanine, glycine, cysteine, serine, arginine, histidine, proline, glutamine, glutamate, methionine, and valine), ketogenic (lysine and leucine) and those that are both glucogenic and ketogenic (tyrosine, phenylalanine, threonine, tryptophan and isoleucine). Glucogenic amino acids can be transformed into intermediates of the TCA cycle or glucose metabolism for the synthesis of glucose through gluconeogenesis (Owen et al., 2002). Ketogenic amino acids can be degraded into ketone bodies and acetyl-CoA. Figure 2 shows that the *I. scapularis* genome contains the enzymes that allows a full degradation of both glucogenic and ketogenic amino acids.

**Figure 3:**
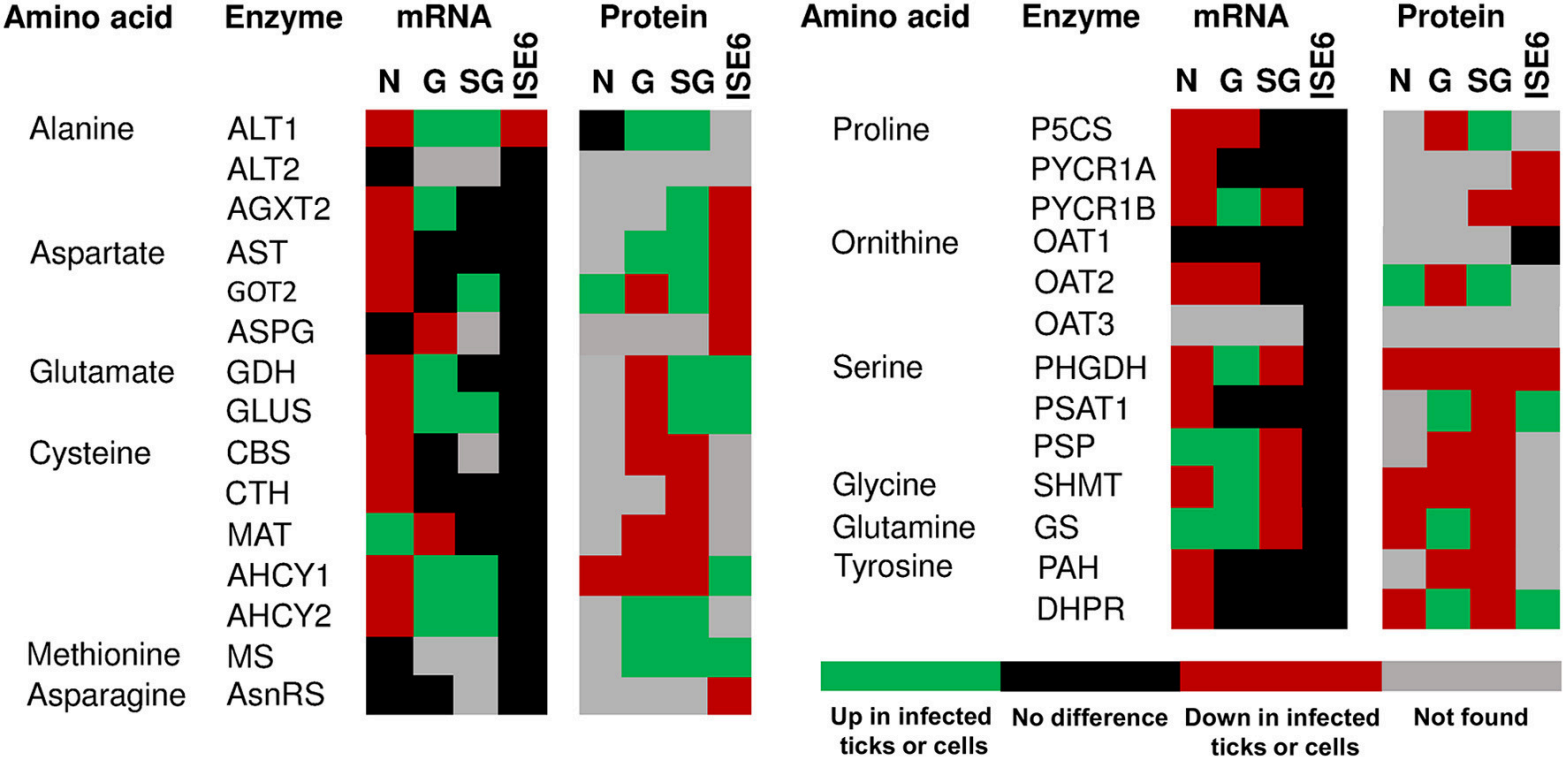
mRNA and protein levels of the *I. scapularis* amino acid synthesis enzymes in response to *A. phagocytophilum* infection. Comparison of amino acid synthesis enzymes mRNA and protein levels in *I. scapularis* nymphs (N), female midguts (G), female salivary glands (SG) and ISE6 cells (ISE6) in response to *A. phagocytophilum* infection. Transcriptomics and proteomics data were obtained from previously published datasets available on the Dryad repository database, NCBI's Gene Expression Omnibus database and ProteomeXchange Consortium via the PRIDE partner repository with the dataset identifier PXD002181 and doi: 10.6019/PXD002181 (Ayllón et al., 2015; Villar et al., 2015). Name of enzymes were abbreviated as in Table 1 and Figure 1.

**Figure 4:**
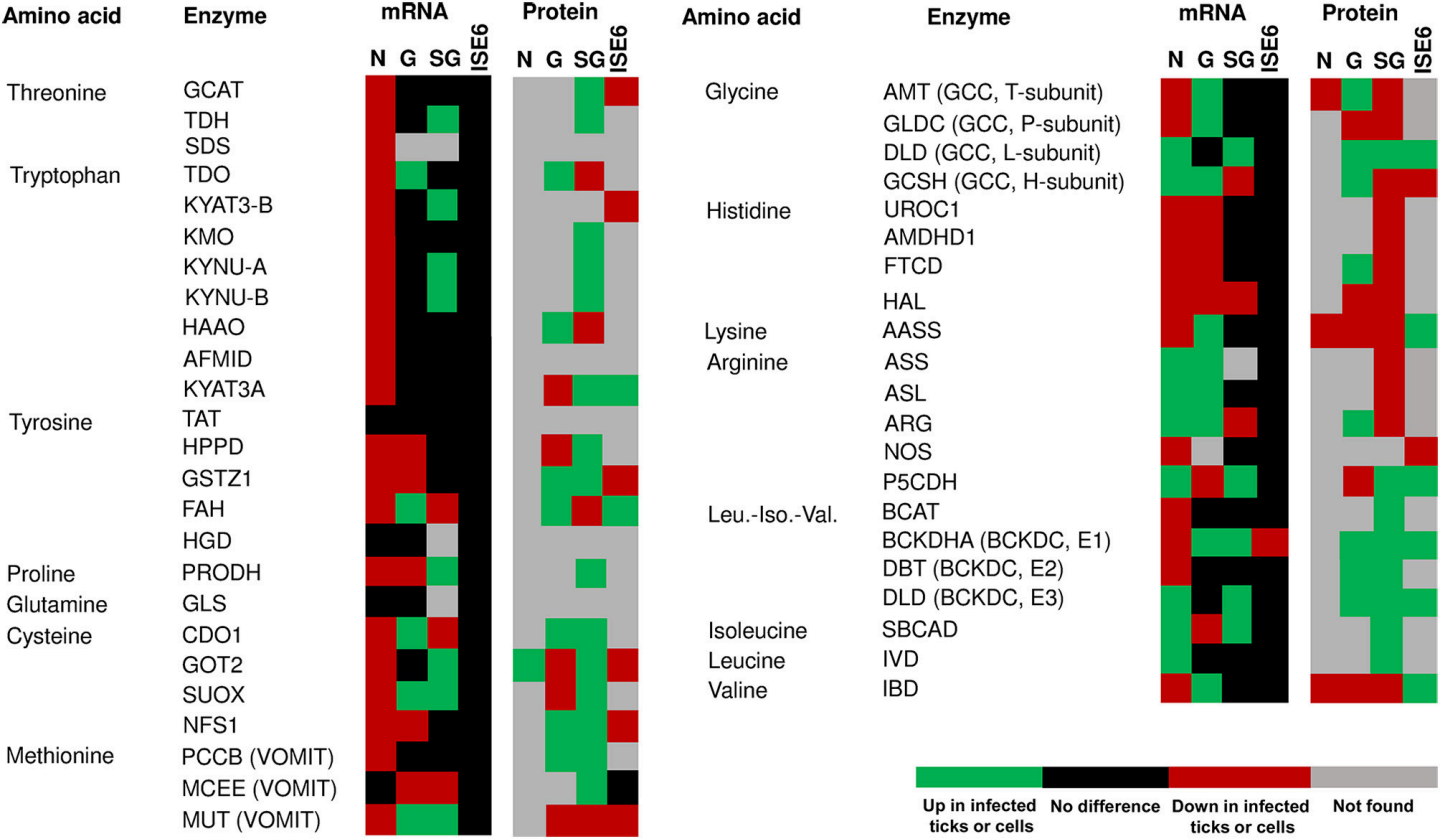
mRNA and protein levels of the *I. scapularis* amino acid degradation enzymes in response to *A. phagocytophilum* infection. Comparison of amino acid degradation enzymes mRNA and protein levels in *I. scapularis* nymphs (N), female midguts (G), female salivary glands (SG) and ISE6 cells (ISE6) in response to *A. phagocytophilum* infection. Transcriptomics and proteomics data were obtained from previously published datasets available on the Dryad repository database, NCBI's Gene Expression Omnibus database and ProteomeXchange Consortium via the PRIDE partner repository with the dataset identifier PXD002181 and doi: 10.6019/PXD002181 (Ayllón et al., 2015; Villar et al., 2015). Name of enzymes were abbreviated as in Table 1 and Figure 2.

The amino acid metabolism response to *A. phagocytophilum* infection was characterized using the quantitative transcriptomics and proteomics data generated from uninfected and *A. phagocytophilum*-infected *I. scapularis* ticks and ISE6 cultured cells (Ayllón et al., 2015; Villar et al., 2015). As in previous reports for other biological processes (Ayllón et al., 2015; Villar et al., 2015; Cabezas-Cruz et al., 2016, 2017a,b), most of the identified amino acid metabolism genes were differentially regulated in response to *A. phagocytophilum* infection in at least one of the analyzed tick samples (Figures 3, 4). Thirty-one (43%), 12 (16%), 32 (44%), and 58 (80%) amino acid metabolism components were identified in both transcriptome and proteome of ISE6 cells, nymphs, adult female midguts and salivary glands, respectively (Figures 3, 4). The proteomics results showed that various proteins were not identified in one or several samples (Figures 3, 4), suggesting low protein levels in these cells or tissues. However, the levels of several proteins changed in response to infection (Figures 3, 4). Considering the protein levels to provide an indicator of the effect of *A. phagocytophilum* infection on tick amino acid metabolic pathways, the results showed a global decrease in serine, glycine, glutamine and tyrosine synthesis enzymes in nymphs, and adult midguts and salivary glands (Figure 3). Histidine, lysine and arginine degradation enzymes were underrepresented in salivary glands (Figure 4). On the contrary, leucine, isoleucine and valine degradation enzymes were overrepresented in most tick tissues and ISE6 cells (Figure 4). These results supported the presence of tissue-specific differences in the tick cell response to infection (Sunyakumthorn et al., 2013; Ayllón et al., 2015; Villar et al., 2015; Cabezas-Cruz et al., 2016, 2017a,b). The datasets used in this analysis on the tick transcriptomics and proteomics response to *A. phagocytophilum* infection have been validated before in several studies (Ayllón et al., 2015; Villar et al., 2015; Cabezas-Cruz et al., 2016, 2017a,b).

### *Anaplasma phagocytophilum* infection redirects tick metabolism toward production of phosphoenolpyruvate

**Figure 5:**
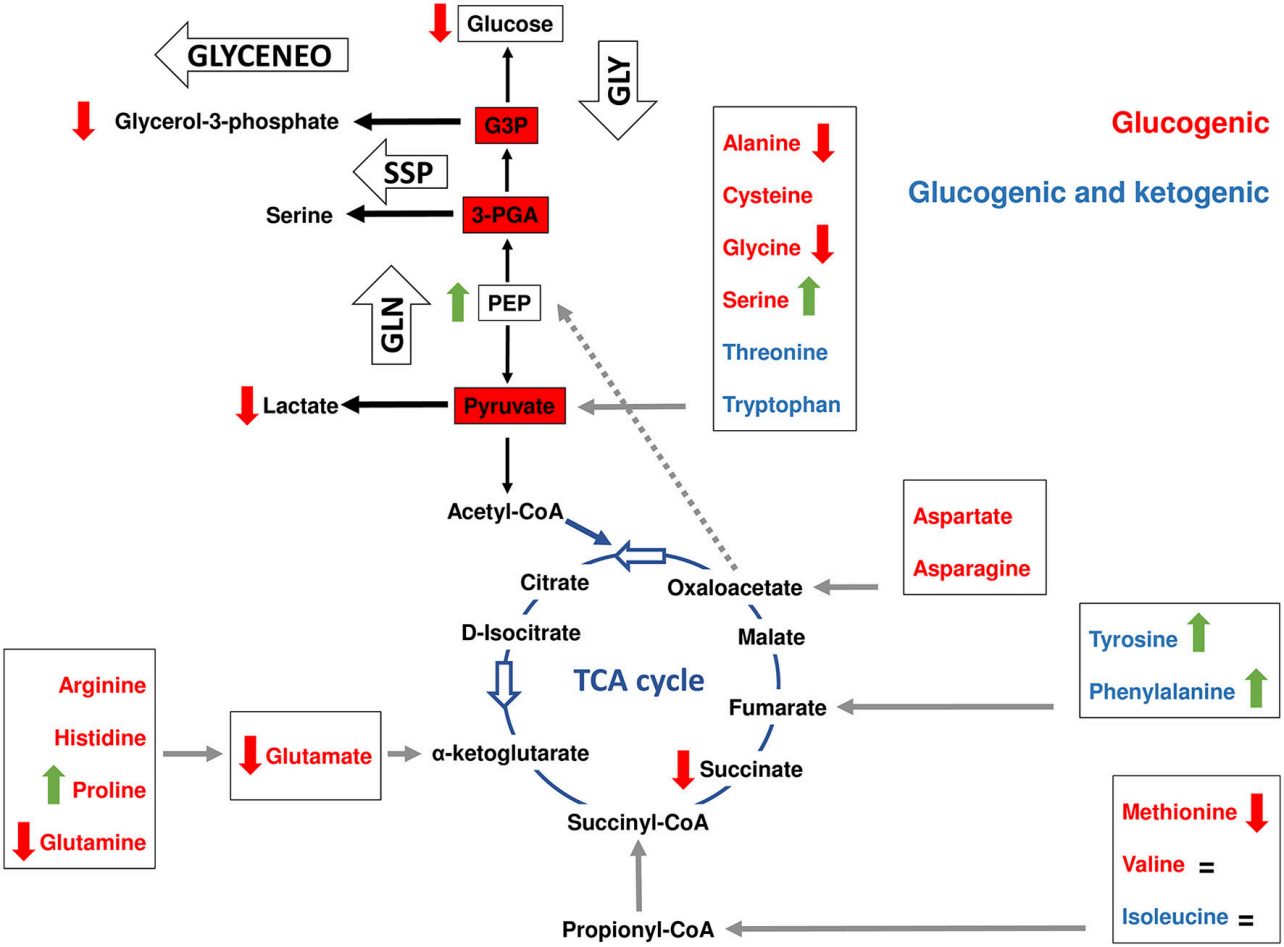
Interplay between amino acid and carbohydrate metabolism in tick ISE6 cells. Simplified gluconeogenesis (GLN), glyceroneogenesis (GLYCENEO), glycolysis (GLY), *de novo* serine synthesis (SSP) and TCA cycle pathways are represented. The amino acids were colored following their classification in glucogenic (red) and those that are both glucogenic and ketogenic (blue). The entry points (gray arrows) of each amino acid group (boxed) to the TCA cycle are shown as well as the amino acids that are transformed to pyruvate. Data on amino acid and metabolic intermediate levels are represented by colored vertical arrows (red decrease, green increase and = no change) was collected from a previous report (Villar et al., 2015), except for phosphoenolpyruvate (PEP) and glycerol-3-phosphate that were measured in the present study. Glyceraldehyde 3-phosphate was abbreviated as G3P.

We previously showed that *A. phagocytophilum* infection activates the expression of glycolytic genes by a HIF-1α-mediated mechanism (Cabezas-Cruz et al., 2017a). Interestingly, *A. phagocytophilum* infection increased the levels of serine, but decreased the levels of alanine (Figure 5). Serine is an allosteric activator of pyruvate kinase (PK) isoform M2 (PKM2), which catalyzes the last step of glycolysis to convert PEP to pyruvate and produce one molecule of ATP (Amelio et al., 2014; Yang and Vousden, 2016). The *I. scapularis* PK shares 64% identity with the human PKM2 and is overrepresented in *A. phagocytophilum*-infected ISE6 cells (Cabezas-Cruz et al., 2017a). In serine deprivation conditions, PKM2 activity is lowered, resulting in diversion of the 3-PGA pool into the *de novo* serine synthesis pathway (SSP) (Yang and Vousden, 2016). In contrast, when serine is abundant, PKM2 is fully activated, allowing the consumption of glucose through aerobic glycolysis (Amelio et al., 2014). In contrast, alanine is an allosteric inhibitor of PK (Gaitán et al., 1983; Berg et al., 2002). The high level of serine and low level of alanine suggested that, in addition to PK overrepresentation in *A. phagocytophilum-*infected ISE6 cells, the activity of PK might be enhanced. An activation of PK activity may increase the consumption of its substrate PEP.

**Figure 6:**
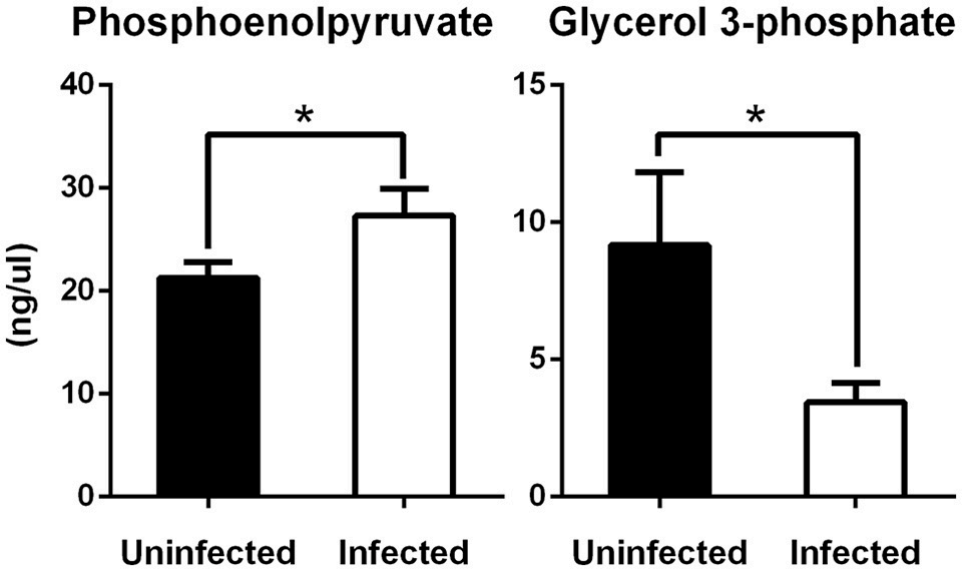
Levels of phosphoenolpyruvate and glycerol 3-phosphate in tick ISE6 cells in response to *A. phagocytophilum* infection. The concentration of phosphoenolpyruvate (PEP) and glycerol 3-phosphate (G-3P) was measured using a colorimetric assay. ISE6 tick cells were inoculated with *A. phagocytophilum* and sampled at 7 days post-infection. G-3P and PEP levels (ng/μl) were compared between untreated and treated cells by Student's *t*-test with unequal variance. Asterisks denote statistical significant differences between groups (*P* < 0.05; *N* = 4).

To evaluate the effect of infection on the levels of PEP, the intracellular concentration of this metabolite was measured by a colorimetric assay, showing that the levels of PEP increased significantly in *A. phagocytophilum*-infected ISE6 cells (Figure 6). Two enzymes are responsible for the production of PEP, the glycolysis enzyme enolase (ENOL) and the gluconeogenesis enzyme phosphoenolpyruvate carboxykinase (PEPCK). The PEP produced by ENOL may be transformed rapidly to pyruvate by PK to keep the glycolytic flow. There are, however, four major pathways in which the PEP produced by PEPCK plays a key role (Yang et al., 2009). These pathways are gluconeogenesis, glyceroneogenesis, the SSP, and the conversion of the carbon skeletons of amino acids to PEP (via PEPCK) and then to pyruvate (via PK) for subsequent oxidation in the TCA cycle as acetyl-CoA (Yang et al., 2009). A suitable hypothesis to explain the increase in PEP is that all the above pathways that use this metabolite are inhibited in *A. phagocytophilum*-infected ISE6 cells.

### *Anaplasma phagocytophilum* infection decreases gluconeogenesis and glyceroneogenesis

All eukaryotes have genes for a mitochondrial (PEPCK-M) and a cytosolic (PEPCK-C) PEPCK (Yang et al., 2009). Both enzymes were found in the genome of *I. scapularis* (Cabezas-Cruz et al., 2017a). As previously reported, *A. phagocytophilum* infection inhibits gluconeogenesis and decreases the concentration of glucose by decreasing the protein levels of PEPCK-C (Villar et al., 2015; Cabezas-Cruz et al., 2017a). Gluconeogenesis and glyceroneogenesis are related pathways and both are regulated by PEPCK-C (Berg et al., 2002; Nye et al., 2008). Glyceroneogenesis is the *de novo* synthesis of glycerol 3-phosphate (G-3P) from precursors other than glucose and glycerol, including pyruvate, lactate, alanine, and TCA cycle anions (Nye et al., 2008). The decrease in the levels of lactate and alanine in *A. phagocytophilum*-infected ISE6 cells (Figure 5) suggested that infection by this pathogen, in addition to gluconeogenesis, also inhibits glyceroneogenesis. To test the effect of *A. phagocytophilum* infection on glyceroneogenesis, the intracellular concentration of G-3P was measured in *A. phagocytophilum*-infected ISE6 cells by a colorimetric assay. The levels of G-3P decreased significantly in infected cells (Figure 6).

Dihydroxyacetone phosphate (DHAP) is transformed into G-3P by the enzyme glycerol-3-phosphate dehydrogenase (GPDH) and DHAP can be produced by fructose-bisphosphate aldolase A (ALDA) and triosephosphate isomerase (TPI). The enzyme ALDA was underrepresented in ISE6 cells and salivary glands infected with *A. phagocytophilum* (Cabezas-Cruz et al., 2017a). Interestingly, it was recently shown that PEP competitively inhibits the interconversion of glyceraldehyde-3-phosphate (G3P) and DHAP by TPI and therefore is considered an inhibitor of TPI (Grüning et al., 2014). Therefore, the increase in the intracellular concentration of PEP may act as an additional mechanism to inhibit glyceroneogenesis.

### *Anaplasma phagocytophilum* infection decreases *De novo* serine synthesis pathway but increases the expression of serine transporters in ISE6 cells

Figure 5 shows that except for valine, serine and proline, *A. phagocytophilum* infection decreased the levels of all glucogenic amino acids found in ISE6 cells (Villar et al., 2015). The levels of valine did not change in response to *A. phagocytophilum* infection, but the levels of serine and proline increased significantly (Figure 5). Despite that the levels of proline increased, the levels of glutamate decreased in response to infection (Figure 5). Proline is considered to be a glucogenic amino acid because it can be converted into glutamate that enters the TCA cycle through α-ketoglutarate. This finding suggested that during *A. phagocytophilum* infection, proline has little or no contribution to gluconeogenesis.

Furthermore, while the levels of serine increased, the enzyme 3-phosphoglycerate dehydrogenase (PHGDH) that catalyzes the first step in SSP, was underrepresented in ISE6 cells, nymphs, midguts and salivary glands (Figure 3). The enzyme PHGDH plays a major role in redirecting the glycolytic intermediate 3-PGA to SSP (Amelio et al., 2014; Samanta and Semenza, 2016). In addition, phosphoserine aminotransferase (PSAT1), the second enzyme of SSP, uses glutamate in a transamination reaction that converts 3-phosphohydroxypyruvate to phosphoserine. As mentioned above, glutamate decreased in infected cells, which suggested that the reaction catalyzed by PSAT1 may be hampered by the low levels of this amino donor. Serine can also be derived from glycine by action of the enzyme serine hydroxymethyltransferase (SHMT), which catalyzes the interconversion of glicine and serine. SHMT was underrepresented in nymphs, midguts and salivary glands and was not found in infected ISE6 cells, but the concentration of glycine was found to decrease in *A. phagocytophilum*-infected ISE6 cells (Figures 3, 5). Therefore, the increase in intracellular serine levels in infected cells cannot be explained by SSP or the activity of SHMT. An alternative explanation is that *A. phagocytophilum* induces a higher rate of serine uptake through membrane transporters (Yang and Vousden, 2016). Two serine transporters were found in the genome of *I. scapularis* (accession numbers ISCW015439 and ISCW017507). Both transporters were overrepresented in infected salivary glands and ISE6 cells (data not shown), which suggests that serine is taken from the extracellular milieu during *A. phagocytophilum* infection in tick cells.

### Contribution of phenylalanine and tyrosine to the OAA/PEPCK-M/PEP node in *A. phagocytophilum*-infected ISE6 cells

As mentioned above, *A. phagocytophilum* infection increased the intracellular concentration of PEP and inhibited the pathways that use this metabolite downstream gluconeogenesis (i.e., gluconeogenesis, glyceroneogenesis and SSP). PEP (via PEPCK) is also involved in the cycling of amino acids to PEP and then to pyruvate (via PK) for subsequent oxidation in the TCA cycle as acetyl-CoA (Yang et al., 2009). While PEPCK-C was underrepresented in *A. phagocytophilum*-infected ISE6 cells (Villar et al., 2015; Cabezas-Cruz et al., 2017a), one of the PEPCK-M isoforms found in the *I. scapularis* genome was upregulated and overrepresented in *A. phagocytophilum*-infected ISE6 cells (Cabezas-Cruz et al., 2017a). PEPCK-M cannot replace PEPCK-C in gluconeogenesis (Méndez-Lucas et al., 2013). However, transformation of oxaloacetate (OAA) into PEP from the mitochondria via PEPCK-M can contribute up to 40% of the cytosolic PEP pool (Stark et al., 2009; Yang et al., 2009).

Glucogenic and ketogenic amino acids can enter the TCA cycle at different points and be transformed into OAA, which is the substrate of PEPCK-M to produce PEP. Arginine, histidine, proline, and glutamine are transformed into glutamate, which enters the TCA cycle through α-ketoglutarate (Figure 5). Despite that some of the enzymes that transform glutamate into α-ketoglutarate were overrepresented (i.e., GDH and PSAT1) (Figure 3), as previously mentioned the concentration of glutamate decreased in *A. phagocytophilum*-infected ISE6 cells (Figure 5), suggesting that arginine, histidine, proline and glutamine have a small contribution to the TCA cycle and OAA production.

Another entry point of amino acids (i.e., methionine, valine, threonine, and isoleucine) to the TCA cycle is succinyl-CoA, which results from the VOMIT pathway (Figure 2). These four amino acids were not affected (valine and isoleucine), decreased (methionine) or were not found (threonine) in *A. phagocytophilum*-infected ISE6 cells (Figure 5). In addition, the last enzyme of the VOMIT pathway, methylmalonyl-CoA mutase, was underrepresented in *A. phagocytophilum*-infected ISE6 cells (Figure 4). These findings suggested that the contribution of amino acids to the succinyl-CoA pool during *A. phagocytophilum* infection is limited. This was supported by the observation that the levels of succinate, the TCA cycle intermediate that results from succinyl-CoA, decreased in infected ISE6 cells (Villar et al., 2015).

As shown in Figure 1, the interconversion of aspartate and asparagine is achieved by the enzymatic activity of AsnRS (aspartate to asparagine) and ASPG (asparagine to aspartate). Both enzymes were underrepresented in *A. phagocytophilum*-infected ISE6 cells (Figure 3). Aspartate can be transformed into glutamate by AST, an enzymatic reaction that also produces OAA. As shown in Figure 3, aspartate aminotransferase (AST) was also underrepresented in *A. phagocytophilum*-infected ISE6 cells. Despite that aspartate and asparagine were not found in uninfected or *A. phagocytophilum*-infected ISE6 cells (Villar et al., 2015), the low protein levels of the enzymes involved in their metabolism suggested that these two amino acids do not contribute to OAA production.

**Figure 7:**
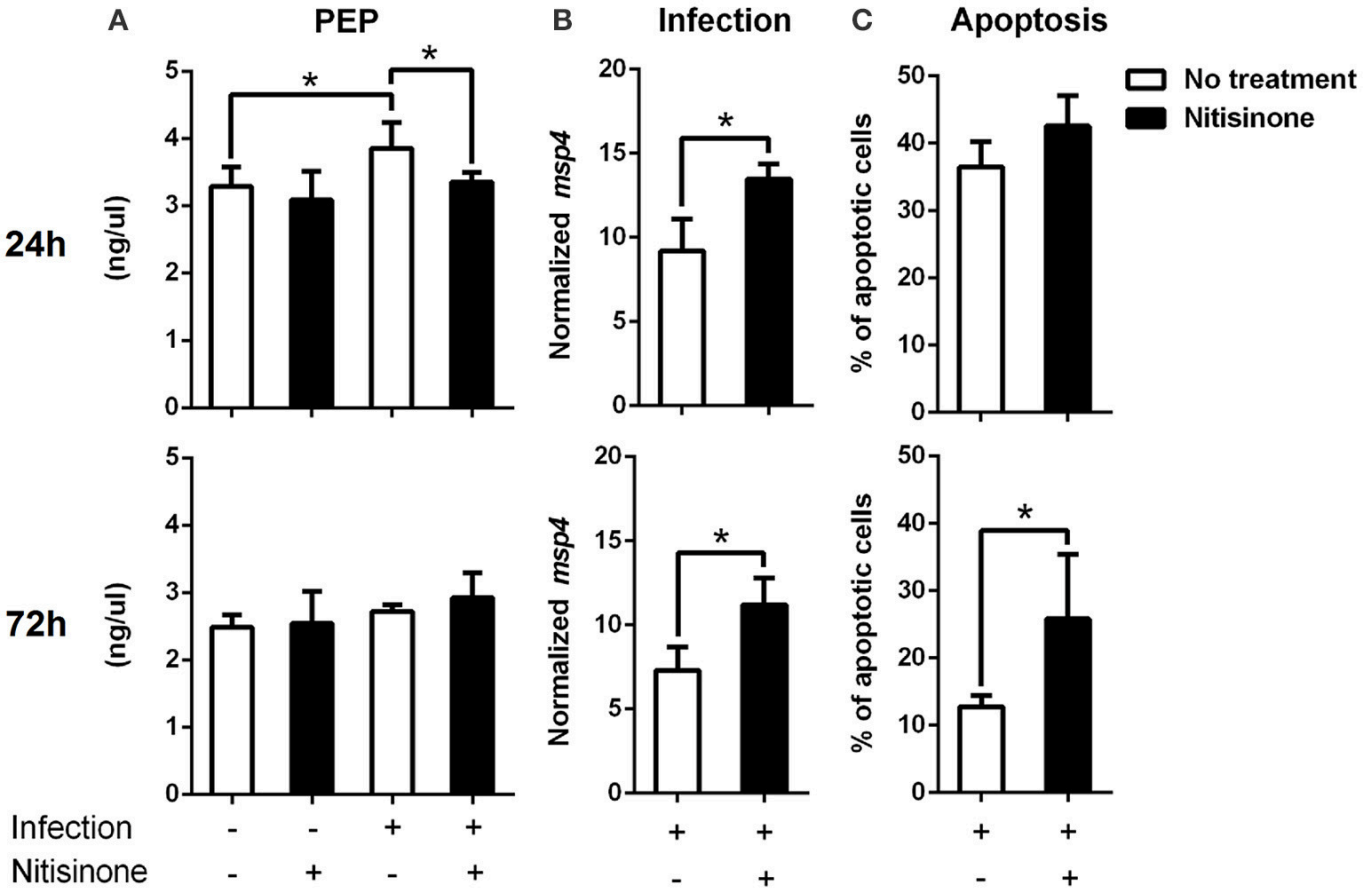
Inhibition of the tyrosine degradation pathway by Nitisinone in *A. phagocytophilum*-infected ISE6 cells. **(A)** ISE6 tick cells were inoculated with *A. phagocytophilum* and sampled at 24 and 72 h post-infection. The concentration of PEP was measured using a colorimetric assay. PEP levels (ng/μl) were compared between untreated and treated cells with Nitisinone to inhibit the activity of HPPD, by Student's *t*-test with unequal variance. **(B)** *A. phagocytophilum* DNA levels were determined in infected ISE6 tick cells untreated or treated with Nitisinone. Bacterial DNA levels were determined by *msp4* real-time PCR normalizing against tick *rps4*. Results are shown as Ave+SD normalized Ct values and were compared between untreated and treated cells by Student's *t*-test with unequal variance. **(C)** The percent (%) of apoptotic cells was determined by flow cytometry in uninfected ISE6 tick cells untreated or treated with Nitisininone. Results were represented as Ave+SD and compared between untreated and treated cells by Student's *t*-test with unequal variance. Asterisks denote statistical significant differences between groups (*P* < 0.05; *N* = 4).

Finally, the degradation of tyrosine and phenylalanine produces fumarate, and the levels of these two amino acids were higher in *A. phagocytophilum*-infected ISE6 cells (Figures 2, 5). The enzyme DHPR, which converts tetrahydrobiopterin into dyhydrobiopterin, a cofactor necessary for the transformation of phenylalanine into tyrosine, was overrepresented in *A. phagocytophilum*-infected ISE6 cells (Figure 3). The last enzyme of the tyrosine degradation pathway, FAH, produces fumarate and acetoacetate and was overrepresented in *A. phagocytophilum*-infected ISE6 cells (Figure 4). These results suggested that the increase of PEP in *A. phagocytophilum*-infected ISE6 cells was due to the transformation of tyrosine to PEP via TCA cycle and PEPCK-M. To test this hypothesis *A. phagocytophilum*-infected and uninfected ISE6 cells were treated with Nitisinone, an inhibitor of HPPD which is the second enzyme of the tyrosine degradation pathway (Figure 2). In agreement with our hypothesis, Nitisinone inhibited the increase of PEP levels induced by *A. phagocytophilum* infection in ISE6 cells (Figure 7A). The effect of Nitisinone on PEP levels was significant during early (24 h) but not late (72 h) *A. phagocytophilum* infection of ISE6 cells (Figure 7A). Interestingly, Nitisinone treatment increased the bacterial burden during early and late *A. phagocytophilum* infection (Figure 7B). Finally, we measured the percentage of apoptotic cells after Nitisinone treatment in *A. phagocytophilum*-infected ISE6 cells. After 72 h, the Nitisinone treatment increased significantly the apoptosis in *A. phagocytophilum*-infected ISE6 cells (Figure 7C).

## Discussion

The metabolic crosstalk between hosts and pathogens is more complex than previously realized (Olive and Sassetti, 2016). It was initially thought that the adaptation to a pathogenic lifestyle was exclusively associated to the acquisition of traits to overcome host immunity. However, recent evidences have uncovered that adaptation of pathogens to host metabolism and host metabolism manipulation by pathogens are as important as overcoming host immunity (Olive and Sassetti, 2016). For intracellular pathogens that have to compete for nutrients within the host cells, metabolism is a keystone to the outcome of infection. Several recent studies show that amino acids are central to the host-pathogen metabolic interaction (Belland et al., 2003; Baruch et al., 2014; Olive and Sassetti, 2016; Østergaard et al., 2016). For example, group A *Streptococcus* (GAS) uses asparagine for sensing the host immune status (Baruch et al., 2014). Toxins secreted by GAS trigger endoplasmic reticulum stress response which upregulates asparagine synthetase leading to higher levels of host-derived asparagine. This asparagine then activates the streptococcal invasion locus, which controls genes involved in virulence, growth and metabolism (Baruch et al., 2014). Therefore, it appears that GAS senses the proximity to host cells by monitoring the host response to bacterial toxins (Baruch et al., 2014; Olive and Sassetti, 2016).

An *in vivo* study of *Salmonella enterica* serovar Typhimurium (hereafter *S*. Typhimurium) in mouse revealed that pathogen infection induces a global differential gene expression (Liu et al., 2010). During early infection, *S*. Typhimurium produced a complete shut-off of the oxidative phosphorylation genes, while at a later stage of infection branched chain amino acids (valine, leucine and isoleucine) genes were significantly downregulated by the infection (Liu et al., 2010). Two examples of pathogen dependency on host metabolic status are *Francisella tularensis* and *Mycobacterium tuberculosis*, which are facultative and obligate intracellular pathogens, respectively (Olive and Sassetti, 2016). These pathogens require *de novo* tryptophan synthesis during infection to circumvent the depletion of host tryptophan that occurs during active T cell responses (Chu et al., 2011; Zhang et al., 2013).

One of the best-studied examples of bacterial response to host amino acid modulation is *Chlamydia trachomatis*. This obligate intracellular pathogen has lost the ability to produce several amino acids including tryptophan that it scavenges from the host (Zhang and Rubin, 2013; Olive and Sassetti, 2016). In humans, interferon-γ (IFNγ) induces a specific antimicrobial response through the induction of indoleamine-2,3-dioxegenase (IDO) which in turn depletes local stores of tryptophan (Pfefferkorn, 1984). *Chlamydia trachomatis* senses IDO-mediated tryptophan depletion and responds by differentiating to a viable but non-replicating form which allows the pathogen to cause long-term persistent infections even in the face of ongoing immune responses (Olive and Sassetti, 2016). The presence of an inducible partial tryptophan operon enables *C. trachomatis* to use indole to synthetize tryptophan even in the presence of high levels of the tryptophan-degrading enzyme IDO (Wood et al., 2003). Once the immune response resolves, IFNγ levels decrease, tryptophan accumulates and *C. trachomatis* reverts to active replication (Belland et al., 2003).

Phosphoenolpyruvate (PEP) also plays an important role in host-bacteria interactions. Most studies on the role of PEP in host-bacteria interactions focus in the PEP Phosphotransferase System (PEP-PTS) which is used by many gram-positive and gram-negative bacteria to uptake carbohydrates (Postma et al., 1993; Barabote and Saier, 2005; Khajanchi et al., 2015; Wang et al., 2015; Antunes et al., 2016). Bacterial PEP-PTS uses PEP as the phosphoryl donor for carbohydrate phosphorylation (Postma et al., 1993). Notably, *Borrelia burgdorferi* lacking one of the PEP-PTS components was unable to establish infection in mice by either needle inoculation or tick transmission (Khajanchi et al., 2015). However, the genomes of several Rickettsiales including *Anaplasma marginale* (closely related to *A. phagocytophilum*), *Rickettsia* spp. and *Wolbachia* sp. lack genes encoding identifiable PTS protein homologs (Barabote and Saier, 2005). This suggests that carbohydrate transport and phosphorylation might not be one of the main roles of PEP in Tick-*A. phagocytophilum* interactions.

**Figure 8:**
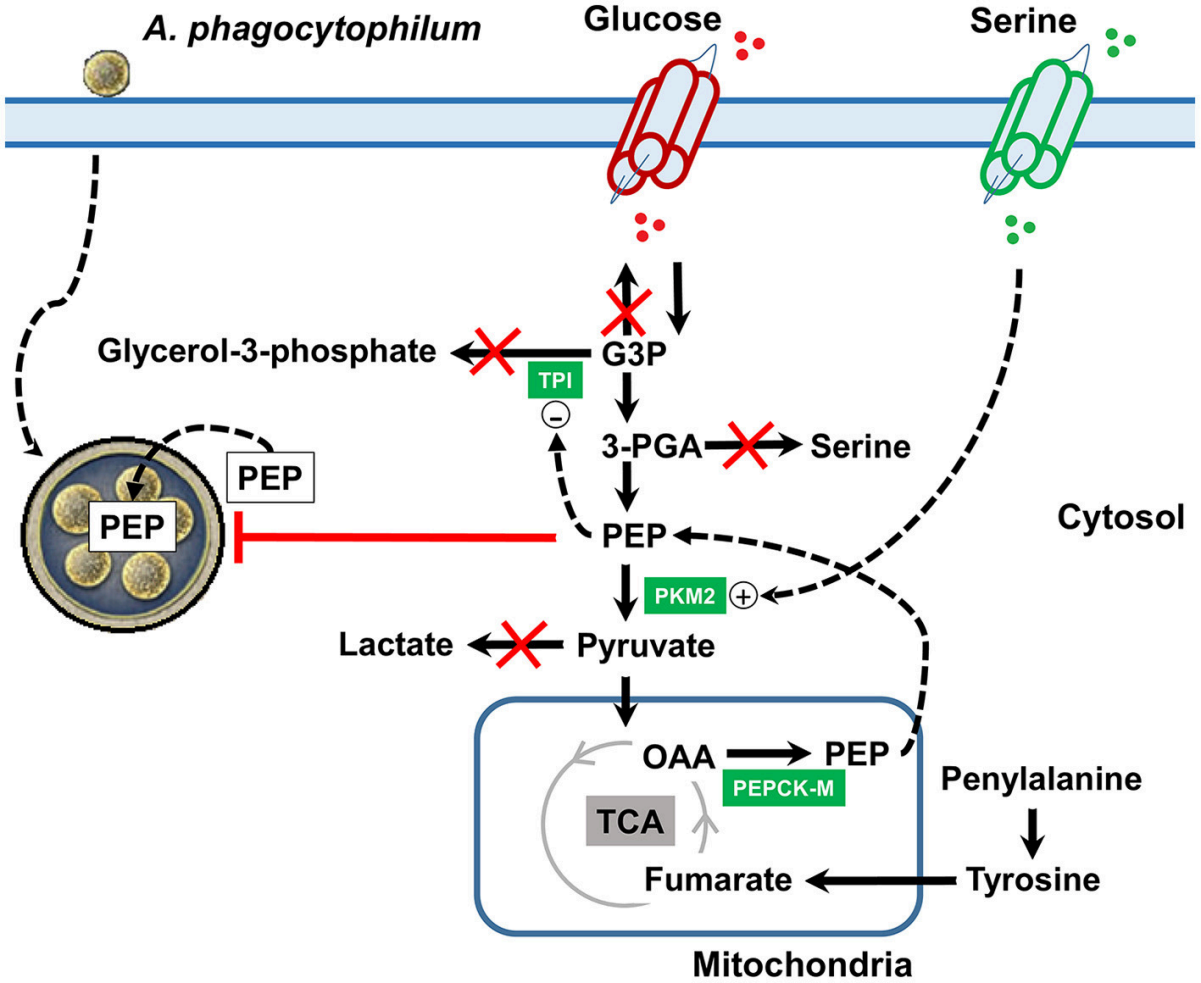
Mechanistic model of metabolic modifications induced by *A. phagocytophilum* infection in tick ISE6 cells. *Anaplasma phagocytophilum* infection increases the protein levels of PKM2 and PEPCK-M in ISE6 cells. TPI is overrepresented in nymphs, midguts and salivary glands, but was not found in ISE6 cells. PEPCK-M transforms mitochondrial OAA into PEP. When the tyrosine degradation pathway (i.e., the enzyme HPPD) was inhibited using Nitisinone, the levels of PEP did not increase in response to *A. phagocytophilum* infection. This strongly suggested that the *A. phagocytophilum*-induced increase in PEP levels is due to activation of the tyrosine/OAA/PEPCK-M/PEP node. Simultaneously, *A. phagocytophilum* infection inhibits glyceroneogenesis (leading to glycerol-3-phosphate), *de novo* serine synthesis pathway (leading to serine) and gluconeogenesis (leading to glucose). PEP is an allosteric inhibitor of TPI and the increase in the levels of PEP may contribute to the inhibition of glyceroneogenesis. Despite that *de novo* serine synthesis pathway is inhibited the levels of serine increased which may be explained by the upregulation of serine transporters located at the cell membrane. Serine is an allosteric activator of PKM2 and the increase in the levels of serine may contribute to the activation of glycolysis. During *A. phagocytophilum* infection, the levels of lactate decrease suggesting that the end product of glycolysis (i.e., pyruvate) is not transformed into lactate, but enters the mitochondria. By this metabolic rearrangement, *A. phagocytophilum* infection increases the cytoplasmic PEP pool which may facilitate the transport of this metabolite inside the parasitophorous vacuole, but at the same time controls the bacterial burden. Name of enzymes were abbreviated as in Table 1.

The results of our study showed that *A. phagocytophilum*, an obligate intracellular bacterium, increases the intracellular concentration of PEP which in turn controls bacterial burden. Remarkably, *A. phagocytophilum* increases PEP synthesis by using tyrosine as carbon source. These results suggested a mechanism by which *A. phagocytophilum* infection induces changes at the crosstalk between carbohydrate and amino acid metabolism in the tick vector *I. scapularis* (Figure 8). Sequestering host PEP may be critical for this bacterium because its genome lacks the enzymes to actively carry out glycolysis to produce PEP (Dunning et al., 2006), but at the same time high concentration of PEP appears to be deleterious for *A. phagocytophilum*. This was revealed by the fact that high levels of PEP concurred with a lower *A. phagocytophilum* burden, while steady levels of PEP increased *A. phagocytophilum* burden (Figures 7A,B). It was previously shown that *A. phagocytophilum* inhibits gluconeogenesis by decreasing the protein levels of cytosolic PEPCK (PEPCK-C), which catalyzes the commitment step of gluconeogenesis (Villar et al., 2015). The inhibition of gluconeogenesis induced by *A. phagocytophilum* infection was confirmed by metabolomics analysis that showed lower levels of glucose in infected ISE6 cells (Villar et al., 2015). In contrast, PEPCK-M was upregulated and overrepresented in infected ISE6 cells (Cabezas-Cruz et al., 2017a). PEPCK-M does not replace the role of PEPCK-C in gluconeogenesis (Méndez-Lucas et al., 2013), but contributes up to 40% of the cytosolic PEP pool (Stark et al., 2009; Yang et al., 2009). Activation of *I. scapularis* PEPCK-C induced apoptosis and reduction of *A. phagocytophilum* levels in ISE6 cells (Villar et al., 2015). Therefore, by downregulation/underrepresentation of PEPCK-C and upregulation/overrepresentation of PEPCK-M, *A. phagocytophilum* infection increases the intracellular levels of PEP without activating gluconeogenesis that is highly detrimental for the pathogen.

That tick cells use tyrosine as a fuel to synthetize PEP during early *A. phagocytophilum* infection (Figure 7A) is an appealing hypothesis because it was recently shown that tyrosine accumulation is lethal for blood feeders, such as kissing bugs, mosquitoes, and ticks (Kopáček and Perner, 2016; Sterkel et al., 2016). Thus, the capacity to avoid very high levels of tyrosine is an essential metabolic adaptation to hematophagy (Kopáček and Perner, 2016; Sterkel et al., 2016). Therefore, by activating the tyrosine/OAA/PEPCK-M/PEP node, *A. phagocytophilum* infection may decrease the tyrosine pool which in turn protects the tick host against tyrosine-induced toxicity. In agreement with this hypothesis, here we found that *A. phagocytophilum* infection increases the protein levels of enzymes involved in tyrosine degradation (i.e., HPPD and GSTZ1 in salivary glands, GSTZ1 and FAH in midguts, and FAH in ISE6 cells; Figure 4) and decreases the enzymes involved in tyrosine synthesis (i.e., PAH in salivary glands, and PAH and DHPR in midguts; Figure 3) in a tissue-specific manner.

## Conclusions

In summary, *A. phagocytophilum* infection increased the concentration of PEP by shunting tyrosine into the TCA cycle, which should increase the concentration of OAA that will be transformed into PEP by PEPCK-M (Figure 8). Further studies with radio labeled tyrosine should assess whether tyrosine carbons are truly recycled to PEP during *A. phagocytophilum* infection. Sequestering host PEP may be critical for this bacterium because it cannot actively carry out glycolysis to produce PEP. However, as shown here, high concentration of PEP appears to be deleterious for *A. phagocytophilum*. These results provide a more comprehensive view of the major amino acid metabolic pathways involved in the response to pathogen infection in ticks, and provides the basis for further studies to develop novel strategies for the control of human granulocytic anaplasmosis by targeting some of the enzymes involved in the tyrosine/OAA/PEPCK-M/PEP node.

## Author contributions

ACC and JF conceived the study. PJE and PA performed the experiments. ACC, PA, DAO, PJE, and JF performed data analyses. ACC and JF wrote the paper, and other co-authors made additional suggestions and approved the manuscript.

### Conflict of interest statement

The authors declare that the research was conducted in the absence of any commercial or financial relationships that could be construed as a potential conflict of interest.
